# Evaluation of cognitive, functional, and behavioral effects observed in EMERGE, a phase 3 trial of aducanumab in people with early Alzheimer's disease

**DOI:** 10.1002/alz.70224

**Published:** 2025-06-22

**Authors:** Jeffrey Cummings, Sharon Cohen, Jennifer Murphy, Holly M. Brothers, Mina Nejati, Fiona Forrestal, Carl de Moor, John O'Gorman, John Harrison, Judith Jaeger, Catherine Jane Mummery, Anton P. Porsteinsson, Michele Potashman, Ying Tian, Lili Yang, Ping He, Samantha Budd Haeberlein

**Affiliations:** ^1^ University of Las Vegas, UNLV Las Vegas NV USA; ^2^ Toronto Memory Program Toronto ON Canada; ^3^ Biogen Cambridge Massachusetts USA; ^4^ Alzheimercentrum AUmC Amsterdam the Netherlands; ^5^ Scottish Brain Sciences Edinburgh UK; ^6^ Institute of Psychiatry Psychology & Neuroscience King's College London London UK; ^7^ CognitionMetrics LLC Stamford Connecticut USA; ^8^ Albert Einstein College of Medicine Bronx New York USA; ^9^ Dementia Research Centre Queen Square Institute of Neurology University College London London UK; ^10^ University of Rochester School of Medicine and Dentistry Rochester New York USA

**Keywords:** Alzheimer's disease, anti‐Aβ mAb, behavioral symptoms, clinical meaningfulness, cognitive decline, functional independence, treatment effect

## Abstract

**INTRODUCTION:**

In EMERGE (NCT02484547), participants receiving aducanumab had significantly less progression versus placebo on all prespecified clinical endpoints at week 78. Here, we explicate the clinical meaningfulness of these treatment effects by analyzing item‐level data and the persistence of treatment benefit.

**METHODS:**

Participants with early Alzheimer's disease (AD) were stratified by apolipoprotein E (*APOE*) ε4 status and randomized (1:1:1) to receive low‐ or high‐dose aducanumab, or placebo. Prespecified principal component analyses (PCAs) per the Statistical Analysis Plan were followed by post hoc examination of individual domains/items across all five clinical endpoints. Progression analysis assessed reduction in clinical decline.

**RESULTS:**

High‐dose aducanumab demonstrated clinically meaningful slowing of progression across clinical endpoints measuring cognition, daily function, and behavioral symptoms. Delay of progression over 18 months was consistent across measures; treatment effects increased over time.

**DISCUSSION:**

Across multiple analyses aducanumab slowed cognitive decline, prolonged functional independence, and attenuated behavioral symptoms in participants with early AD. These outcomes comprise the elements of a clinically meaningful response to treatment.

**Highlights:**

- Endpoints in EMERGE assessed different aspects of cognition, daily function, and behavioral symptoms.
- Treatment benefits were observed across subdomains on all five clinical endpoints.
- Aducanumab meaningfully slowed disease progression in participants with early AD.

## 1 BACKGROUND

Alzheimer's disease (AD) is a progressive neurodegenerative disease that exacts a profound toll on people living with AD and their care partners. Individuals with AD irreversibly lose cognitive abilities and functional independence.^1^ Until the 2021 accelerated approval of aducanumab by the US Food and Drug Administration (FDA), the only approved pharmacological treatment options were symptomatic therapies for patients who had already reached the dementia stage of the disease.^2^ Recent clinical development efforts target the underlying disease pathology in earlier stages, with the goal of delaying disease progression and prolonging independence. Since the 2023 and 2024 FDA traditional approval of lecanemab and donanemab for the treatment of mild cognitive impairment (MCI) and mild dementia due to AD, and its broader availability in the clinical setting, attention has turned to defining and reliably measuring meaningful clinical benefit in ways that translate to a broad group of stakeholders.

Treatment should affect core symptoms of AD and be of a clinically meaningful magnitude. No definitive benchmark exists for defining clinical meaningfulness of therapies that modify the underlying disease process at the MCI and mild dementia stages.^3^ One conventional standard for clinical meaningfulness is demonstrating a drug‐placebo difference on a global measure or a measure of functional abilities.^4^ A combination of measures evaluating cognition, function, behavior, and biomarkers provides a wider perspective on clinical relevance.^3^ The 2024 FDA Draft guidance on Early AD advanced a broader interpretation of a clinically meaningful change–defined as a breadth of domains, depth of particular magnitude, or change in trajectory over time.^5^ While the construct of clinical meaningfulness originates within a regulatory framework, payers, prescribers, and patients desire a deeper understanding of what treatment effects are clinically meaningful.

Consistent treatment effects across tools that measure distinct aspects of the disease progression may help demonstrate a clinically meaningful benefit.^5^ Evidence of ameliorating the underlying pathology of AD by longitudinal biomarker assessments can also provide inferential support for a treatment effect.^5^, ^6^, ^7^ Clinical meaningfulness has been assessed using quantitative approaches, such as the minimal clinically important difference (MCID),^8^ which is the smallest change in a measure that is perceived as beneficial by people living with AD or clinicians. Examination of MCID thresholds has limitations, for example, being influenced by baseline severity, measurement error, and variability across studies. Additional measures, such as patient‐reported outcomes and what matters most (WMM) to patients, have been proposed to capture individual preferences and values of people living with AD and their care partners. A recent study conducted by AD Patient and Caregiver Engagement (AD PACE) found that WMM domains for people with early AD included maintaining independence, social relationships, cognitive abilities, and emotional well‐being.^9^, ^10^ A therapeutic intervention is considered to have a clinically meaningful effect if it can noticeably help people living with AD maintain their cognitive and functional abilities and prolong their independence.^11^, ^12^

In the aducanumab phase 3 studies (EMERGE [NCT02484547] and ENGAGE [NCT02477800]), five well‐established clinical scales measured the broad array of symptoms experienced by participants with early AD. These scales measure concepts identified as important to participants with AD and their care partners.^9^, ^13^

The EMERGE study demonstrated efficacy of aducanumab in participants with early AD.^14^ At the time of trial design, a steering committee external to the trial sponsor and the aducanumab clinical development program recommended that a 25% treatment versus placebo difference on the Clinical Dementia Rating Scale Sum of Boxes (CDR‐SB) over 18 months would be clinically meaningful for participants with early AD.^14^ Following premature trial termination, a 22% treatment difference on the CDR‐SB in favor of high‐dose aducanumab was observed in EMERGE; the treatment effect spans sub‐population comparisons and all five secondary and tertiary clinical endpoints.^14^ Here, we present an evaluation of the treatment effects of aducanumab in EMERGE through a post hoc progression analysis and domain/item‐level analyses of these clinical efficacy endpoints. In addition, we examine the persistence of the treatment effects in the long‐term extension (LTE) period of EMERGE.

## 2 METHODS

### 2.1 Participant population and trial design

In the two phase 3 aducanumab trials, lower cumulative exposure in ENGAGE (109.1 mg/kg) than EMERGE (118.3 mg/kg) due to the implementation of a protocol update resulted in 16.5% less dose‐dependent amyloid plaque reduction on positron emission tomography (PET) and fewer participants reaching amyloid‐negative status (31% in ENGAGE versus 48% in EMERGE).^14^ Reduced amyloid PET is associated with slowing of clinical decline and ENGAGE did not achieve clinical efficacy.^14^ Therefore, we focus here on data from EMERGE.

**FIGURE 1 alz70224-fig-0001:**
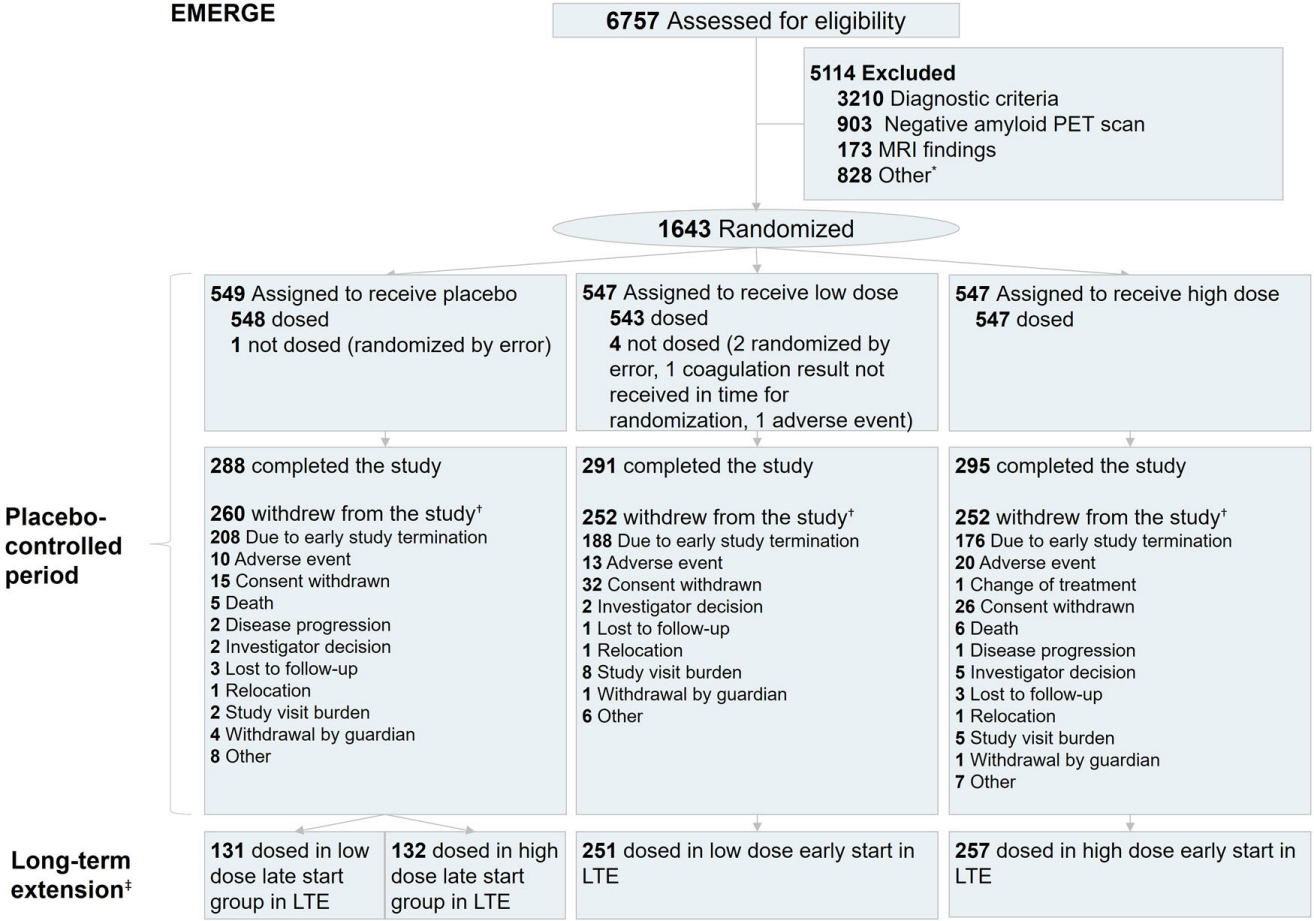
Trial profile. *Other reasons for not meeting inclusion/exclusion criteria include inability to comply with study requirements; presence of diabetes mellitus that, in the judgment of the investigator, cannot be controlled or adequately managed; inability to understand the purpose and risks of the study and provide signed and dated informed consent and authorization to use protected health information in accordance with national and local subject privacy regulations; other unspecified reasons that, in the opinion of the investigator or sponsor, make the individual unsuitable for enrollment; history of or positive test result at screening for hepatitis C virus antibody or hepatitis B virus (defined as positive for both hepatitis B surface antigen AND hepatitis B core antibody); use of allowed chronic medications at doses that have not been stable for at least 4 weeks prior to screening visit 1 and screening up to day 1, or use of Alzheimer's disease (AD) medications at doses that have not been stable for at least 8 weeks. ^†^Some categories with < 1% patients are not displayed, including loss of capacity, pregnancy, and protocol amendment. ^‡^Following the 78‐week placebo‐controlled study period, there was an optional dose‐blind long‐term extension (LTE) period up to week 134. MRI, magnetic resonance imaging; PET, positron emission tomography.

EMERGE was a randomized, double‐blind, placebo‐controlled (PC) trial conducted at 181 sites in 13 countries. Details on the trial design, participant population, and futility analysis have been published^14^ and are summarized in File S1 and Figure 1. Participant recruitment began in September 2015 and ended in July 2018. The high‐dose aducanumab group refers to participants who were titrated to 6 or 10 mg/kg aducanumab, depending on apolipoprotein E (*APOE*) ε4 status and protocol version. Titration to 10 mg/kg was the dose regimen specified in the US product label. 

During the LTE period, participants who were randomized to aducanumab in the double‐blind PC period continued to receive aducanumab at the same dose (early‐start low‐dose or early‐start high‐dose) and participants who were randomized to placebo during the PC period were switched to receive aducanumab (late‐start low‐dose or late‐start high‐dose as randomized at study entry). Early‐start refers to participants who began receiving aducanumab in the PC period, and late‐start refers to participants who began receiving aducanumab in the LTE period. Both the PC and LTE randomizations were stratified by *APOE* ε4 carrier status and study site. Baseline demographics were balanced across early‐ and late‐start high‐dose arms (Table S1 in supporting information).

### 2.2 Outcome measures

The primary endpoint of EMERGE was the drug‐placebo difference in change from baseline CDR‐SB score at week 78. The secondary (Mini‐Mental State Examination [MMSE], Alzheimer's Disease Assessment Scale–Cognitive Subscale [13‐item] [ADAS‐Cog13], Alzheimer's Disease Cooperative Study–Activities of Daily Living Inventory [Mild Cognitive Impairment version] [ADCS‐ADL‐MCI]) and tertiary (Neuropsychiatric Inventory Questionnaire [10‐item version] [NPI‐10]) efficacy outcome measures provide additional important information relevant to considerations of meaningfulness. The CDR‐SB scale has been described by the FDA as a suitable primary outcome measure for early AD and has demonstrated validity and reliability in the longitudinal evaluation of this patient population.^15^ The CDR‐SB is an integrated scale that reflects the observations of an expert clinician following input from care partners and participants with AD. It is a global measure assessing both cognitive effects and daily function and is regarded as a measure of clinically meaningful outcomes.^11^ The ADAS‐Cog13,^16^ the ADCS‐ADL‐MCI,^17^ and the NPI‐10^18^ provide information on cognitive, functional, and neurobehavioral decline, respectively, and are well‐validated and appropriate for evaluating participants with early AD. The MMSE,^19^ though less sensitive in early AD, provides value due to its universal familiarity in assessing dementia.

**RESEARCH IN CONTEXT**

1. **Systematic review**: Traditional definitions of clinically meaningful change in Alzheimer's disease (AD) have relied on data derived from patients with mild to severe stages of AD dementia receiving symptomatic therapy with cholinesterase inhibitors and/or an N‐methyl‐D‐aspartate (NMDA) receptor antagonist. The optimal means of demonstrating clinically meaningful effects for disease‐modifying treatments that target the underlying pathological process of AD, including accumulation of toxic species of amyloid beta (Aβ) and neurofibrillary tangles, remain to be established and require different considerations.
2. **Interpretation**: The effects of treatment with aducanumab meet established and evolving standards for clinical meaningfulness. Data and analyses from the EMERGE study show that aducanumab has a beneficial treatment effect with slowing of decline on a broad range of cognitive, functional, and behavioral measures that reflect disease manifestations important to patients, care partners, and clinicians.
3. **Future directions**: Data from the EMERGE study of aducanumab inform considerations regarding clinical meaningfulness of disease‐modifying therapies.

Each outcome measure was assessed during screening; at weeks 26, 50, and 78; and in the LTE period (weeks 106 and 134; due to early termination of EMERGE, data from beyond week 134 were limited and not formally analyzed). The raters for CDR‐SB and for the secondary endpoints were different assessors (a psychometrician and a CDR‐SB rater) and were independent, blinded to treatment assignment and to status of any amyloid‐related imaging abnormalities (ARIA). See File S2 for a detailed description of each clinical outcome measure.[Fig alz70224-fig-0001]

### 2.3 Statistical analyses

To evaluate the degree of overlap between the endpoints in assessing cognition and function, two principal component analyses (PCAs) were conducted using placebo group data from EMERGE; one PCA was conducted on the baseline item scores of the five clinical endpoints (total of 58 individual items: CDR‐SB, 6; MMSE, 11; ADAS‐Cog13, 13; ADCS‐ADL‐MCI, 18; NPI‐10, 10) and one on the week 78 change from baseline item scores.

Post hoc item‐level analyses in CDR‐SB, MMSE, ADAS‐Cog13, ADCS‐ADL‐MCI, and NPI‐10 as well as care partner distress scales were conducted. A mixed model repeated measures (MMRM) analysis, with fixed effects of treatment, visit, treatment‐by‐visit interaction, baseline score, baseline score–by‐visit interaction, baseline MMSE score, AD symptomatic medication use at baseline, world region, and *APOE* ε4 status (carrier and noncarrier), was performed for each item using the intention to treat (ITT) population. This analysis is considered descriptive as post hoc and multiplicity adjustment was not applied.

In addition to a group‐level analysis examining between‐group changes, a responder‐based analysis examining differing rates of clinically meaningful progression offers a complementary approach to data interpretation with analysis of the odds of progression for individual participants. Please see File S3 for details on the responder analysis and post hoc progression analyses.

Only the high‐dose aducanumab group and the placebo group data from EMERGE are reported here. No notable drug‐placebo differences in clinical measures were observed in the low‐dose group.

## 3 RESULTS

Participant demographic and baseline disease characteristics in EMERGE are summarized in Table 1. Consistent with early symptomatic stages of AD, enrolled participants had the following mean baseline scores (± SD): CDR‐SB score of 2.5 ± 1.0 (scale range 0–18), MMSE score of 26.4 ± 1.8 (scale range 0–30), ADAS‐Cog13 score of 21.9 ± 6.7 (scale range 0–85), ADCS‐ADL‐MCI score of 42.6 ± 5.7 (scale range 0–53), and NPI‐10 score of 4.3 ± 5.9 (scale range 0–120). Disease progression was observed in participants receiving placebo over 78 weeks, with CDR‐SB worsening by 1.74 points, MMSE by 3.3 points, ADAS‐Cog13 by 5.16 points, ADCS‐ADL‐MCI by 4.3 points, and NPI‐10 total score by 1.5 points.^14^

**TABLE 1:** Demographic and baseline disease characteristics in EMERGE.

	Placebo‐controlled period	
	Placebo	High‐dose aducanumab
Characteristics	(*n* = 548)	(*n* = 547)
Age in years, mean ± SD	70.8 ± 7.4	70.6 ± 7.5
Female, *n* (%)	290 (53)	284 (52)
**Race, *n* (%)**		
Asian	47 (9)	42 (8)
White	431 (79)	422 (77)
Education years, mean ± SD	14.5 ± 3.7	14.5 ± 3.6
Alzheimer's disease medications used, *n* (%)	282 (51)	285 (52)
***APOE* ε4, *n* (%)**		
Carriers	368 (67)	365 (67)
Noncarriers	178 (32)	181 (33)
**Clinical stage, *n* (%)**		
MCI due to Alzheimer's disease	446 (81)	438 (80)
Mild Alzheimer's disease	102 (19)	109 (20)
RBANS delayed memory score, mean ± SD	60.5 ± 14.2	60.7 ± 14.2
**CDR global score, *n* (%)**		
0.5	545 (99)	546 (100)
1	3 (1)	1 (< 1)
**ITT**		
CDR‐SB score, mean ± SD	2.5 ± 1.0	2.5 ± 1.1
MMSE score, mean ± SD	26.4 ± 1.8	26.3 ± 1.7
ADAS‐Cog13 score, mean ± SD	21.9 ± 6.7	22.3 ± 7.1
ADCS‐ADL‐MCI score, mean ± SD	42.6 ± 5.7	42.5 ± 5.8
NPI‐10 score, mean ± SD	4.3 ± 5.9	4.5 ± 6.4
OTC	*n* = 313	*n* = 340
CDR‐SB score, mean ± SD	2.5 ± 1.0	2.4 ± 1.0
MMSE score, mean ± SD	26.5 ± 1.8	26.3 ± 1.7
ADAS‐Cog13 score, mean ± SD	22.1 ± 6.7	22.2 ± 7.1
ADCS‐ADL‐MCI score, mean ± SD	42.9 ± 5.1	42.6 ± 5.8

NOTE: Data are mean ± SD or *n* (%).

Abbreviations: ADAS‐Cog13, Alzheimer's Disease Assessment Scale–Cognitive Subscale (13‐item); ADCS‐ADL‐MCI, Alzheimer's Disease Cooperative Study–Activities of Daily Living Inventory (Mild Cognitive Impairment version); *APOE*, apolipoprotein E; CDR‐SB, Clinical Dementia Rating Scale Sum of Boxes; ITT, intention to treat; MCI, mild cognitive impairment; MMSE, Mini‐Mental State Examination; NPI‐10, Neuropsychiatric Inventory Questionnaire (10‐item version); OTC, opportunity to complete week 78 assessment; RBANS, Repeatable Battery for Assessment of Neuropsychological Status; SD, standard deviation.

In the two PCAs (Figure S1 in supporting information) many “components” were needed to explain the variance. Approximately 80% of the variance in the 58 items was explained by 38 components for the baseline item scores and by 31 components for the week 78 change from baseline item scores. Of the 58 items, 66% (baseline) and 58% (week 78) loaded high on a single component, indicating that they measure different aspects of cognition and daily function with little overlap among these items. Aspects that loaded high on the same factor tended to be from the same scale, reflecting internal consistency within scales and limited overlap across scales. Only a small number of components (2 of 38 at baseline and 6 of 31 at week 78) were formed by items from more than one scale. The groupings of items in the results of the baseline item scores and the change from baseline item scores were similar, indicating the robustness of the internal construct of the scales and the inter‐scale relationship.

On the primary endpoint CDR‐SB, an 18‐point scale, placebo declined by 1.74 over 78 weeks. In the aducanumab high‐dose group, the difference versus placebo in mean change from baseline CDR‐SB score at week 78 was −0.39 (*P *= 0.01), representing a 22% slowing in decline with aducanumab.^14^ Slope analysis of the CDR‐SB supported a treatment effect in delaying clinical progression: a difference of −0.30 (*P* = 0.02) in the annual rate of change from baseline (an annual increase of 0.79 points in the aducanumab high‐dose group versus 1.09 points in the placebo group).

**FIGURE 2:**
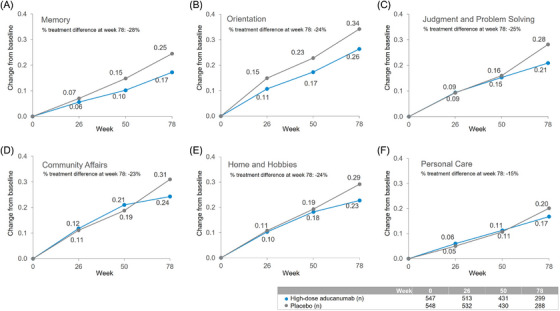
Longitudinal change in each Clinical Dementia Rating Scale Sum of Boxes (CDR‐SB) item in EMERGE: high‐dose aducanumab versus placebo. Adjusted mean change from baseline in each item on the CDR‐SB and at weeks 26, 50, and 78 are plotted with aducanumab high‐dose treatment (blue) and placebo (gray). Higher scores indicate greater impairment. Longitudinal change on (A) Memory (B) Orientation, (C) Judgment and Problem Solving, (D) Community Affairs, (E) Home and Hobbies, and (F) Personal Care are presented here. Percent of treatment differences between high‐dose aducanumab and placebo at week 78 are noted in each panel.

On the CDR‐SB cognitive and functional measures, a drug‐placebo difference in favor of aducanumab was observed across all six domains (Memory, Orientation, Judgment and Problem Solving, Community Affairs, Home and Hobbies, Personal Care), with a reduction in decline of 23% to 28% over 78 weeks from baseline in all three cognitive domains versus placebo (Orientation, 0.26 versus 0.34; Memory, 0.17 versus 0.25; Judgment and Problem Solving, 0.21 versus 0.28) and two functional domains (Community Affairs, 0.24 versus 0.31; Home and Hobbies, 0.23 versus 0.29) (Figure 2). A smaller effect (15% less decline over 78 weeks; 0.17 versus 0.20) was observed in the Personal Care domain (Figure 2F), a domain expected to be less sensitive to change in early AD when there is little compromise in self‐care and little change in an 18‐month observation period. Separation between high‐dose aducanumab versus placebo occurred earlier in two of the three cognitive domains (week 50 for Memory and Orientation) than the functional domains (week 78).

**FIGURE 3:**
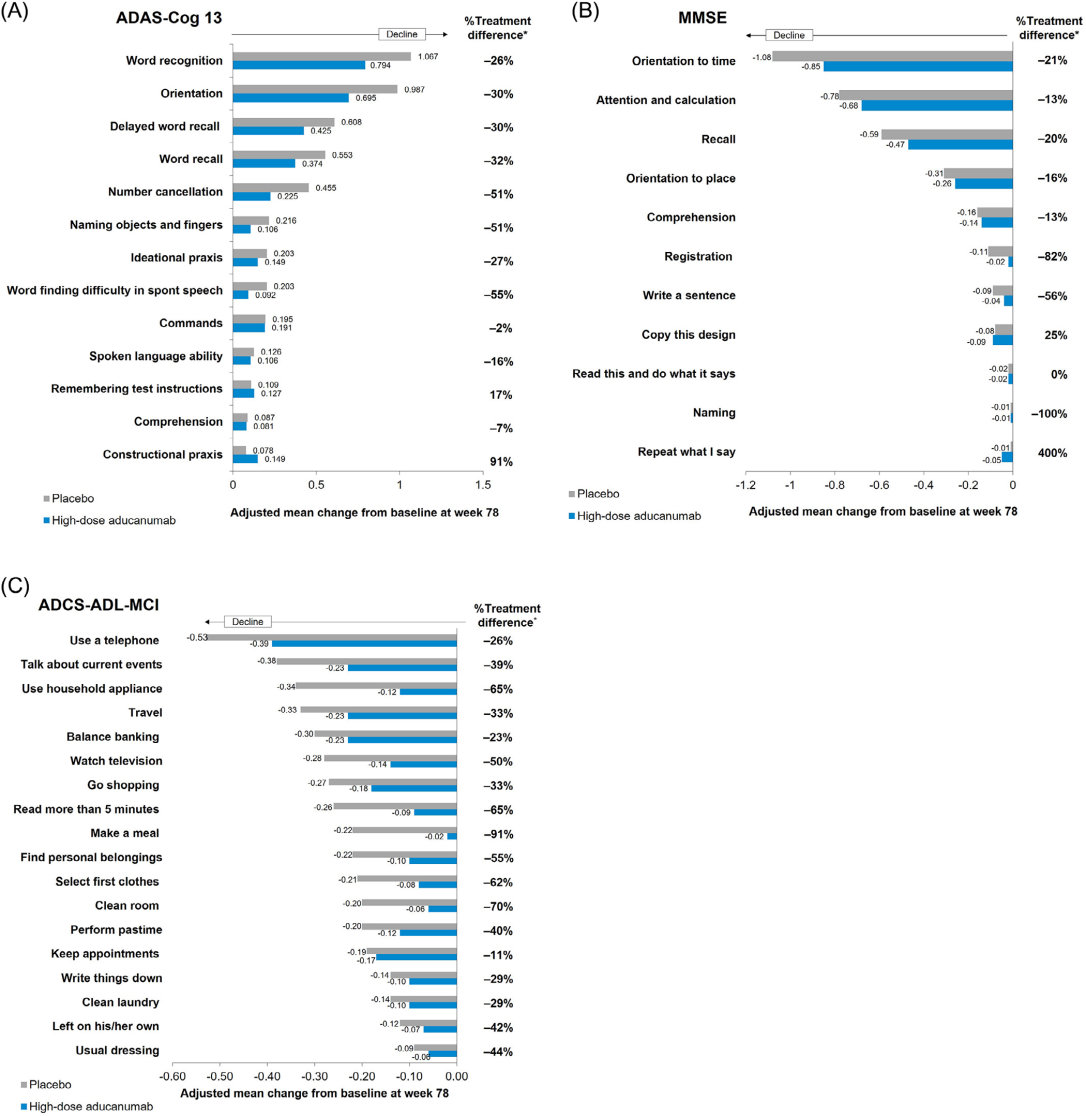
Alzheimer's Disease Assessment Scale–Cognitive Subscale (13‐item) (ADAS‐Cog13), Mini‐Mental State Examination (MMSE), and Alzheimer's Disease Cooperative Study–Activities of Daily Living Inventory (Mild Cognitive Impairment version) (ADCS‐ADL‐MCI) item changes at week 78. Adjusted mean item change from baseline to week 78 in the (A) ADAS‐Cog13 score, (B) MMSE score, and (C) ADCS‐ADL‐MCI score for high‐dose aducanumab (blue) and placebo (gray). On the ADAS‐Cog13, higher scores indicate greater impairment. Scoring range varies for each individual item: Word recognition (0–12), Orientation (0–8), Delayed word recall (0–10), Word recall (0–10), Number cancellation (0–5), Naming objects and fingers (0‐5), Ideation praxis (0–5), Word finding difficulty in spontaneous speech (0–5), Commands (0–5), Spoken language ability (0–5), Remembering test instructions (0–5), Comprehension (0–5), and Constructional praxis (0–5). On the MMSE, lower scores indicate greater impairment. Scoring range varies for each individual item: Orientation to time (0–5), Orientation to place (0–5), Attention and calculation (0–5), Recall (0–3), Comprehension (0–1), Registration (0–3), Write a sentence (0–1), Copy this design (0–1), Read this and do what it says (0–3), Naming (0–2), and Repeat what I say (0–1). On the ADCS‐ADL‐MCI score, lower scores indicate greater impairment. Scoring range varies for each individual item: Finding personal belongings (0–3), Select first clothes (0–3), Usual dressing (0–4), Clean room (0–2), Balance banking (0–2), Write things down (0–2), Clean laundry (0–2), Keep appointments (0–3), Use a telephone (0–4), Make a meal (0–3), Travel (0–3), Talk about current events (0–4), Read more than 5 min (0–3), Watch television (0–3), and Go shopping (0–2); Left on his/her own (0–3), Use household appliance (0–4), and Perform pastime (0–3). Higher scores indicate better ability to perform activities of daily living. ^*^Percent treatment differences between high‐dose aducanumab and placebo are noted for each item.

The ADAS‐Cog13 has 11 items measuring cognition, including Orientation, Word Recall (both immediate and delayed), Word Recognition, and Number Cancellation tests. The decline on these five tasks of the ADAS‐Cog13 was between 26% and 51% less in the high‐dose aducanumab group compared to the placebo group (Figure 3A). On the remaining tasks, there was minimal decline over 78 weeks in the placebo group (Figure 3A).

In the aducanumab high‐dose group, the difference versus placebo in mean change from baseline MMSE score at week 78 was 0.6 (*P *= 0.049), representing an 18% slowing in decline.^14^ On the MMSE, the items assessing Orientation, Attention and Calculation, and Recall exhibited 13% to 21% less decline in the high‐dose aducanumab treated group than in the placebo group (Figure 3B). No notable drug‐placebo differences were observed on the remaining items.

Treatment effects were observed across all 18 items of the ADCS‐ADL‐MCI (Figure 3C). The decline on these items was between 11% and 91% less in the high‐dose aducanumab group compared with the placebo group.

**FIGURE 4:**
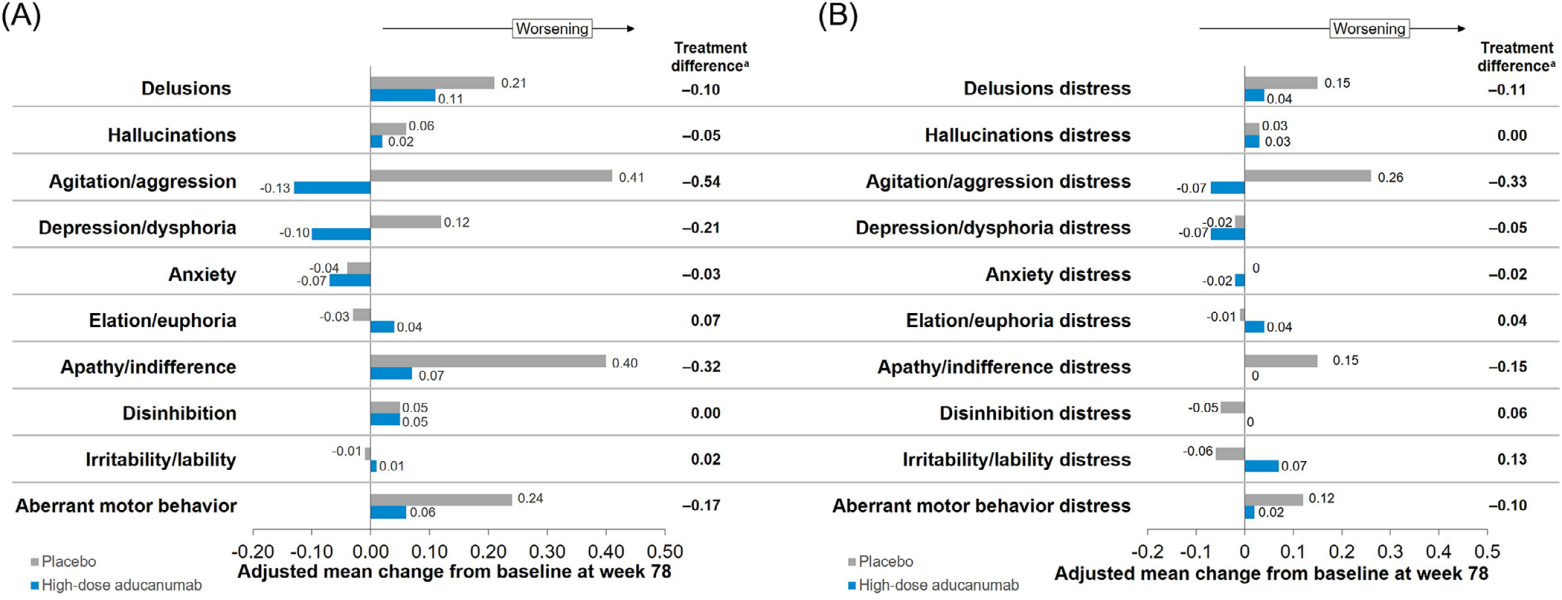
Neuropsychiatric Inventory Questionnaire (NPI‐10) items and caregiver distress in EMERGE: high‐dose aducanumab versus placebo. Adjusted mean NPI‐10 item change from baseline at week 78 (A) and associated caregiver distress score (B). Each item on the NPI‐10 has a subscore between 0 and 12, with higher scores indicative of greater severity. Each corresponding caregiver distress score has a range between 0 and 5. This figure shows high‐dose aducanumab (blue) and placebo (gray) at week 78. *Percent treatment differences between high‐dose aducanumab and placebo are noted in each panel.

Analysis of the NPI‐10 items showed drug‐placebo differences in favor of aducanumab for Agitation/Aggression (−0.54 treatment difference), Apathy/Indifference (−0.32 treatment difference), Depression/Dysphoria (−0.21 treatment difference), Aberrant Motor Behavior (−0.17 treatment difference), and Delusions (−0.10 treatment difference). Smaller treatment differences were seen for Anxiety, Hallucinations, and Irritability. Participants receiving high‐dose aducanumab showed improvement or smaller decline in score changes from baseline over 78 weeks for these symptoms (Figure 4A). The effects were reflected in drug‐placebo differences on associated care partner distress items (84% reduction versus placebo on the overall NPI‐10 care partner distress score, Figure 4B).

Over 78 weeks, the high‐dose aducanumab arm showed slower rates of decline than placebo on the MMSE (difference of 0.6 in mean change from baseline; −18%; *P* < 0.05), ADAS‐Cog13 (difference of −1.40 in mean change from baseline; −27%; *P* = 0.01), and ADCS‐ADL‐MCI (difference of 1.7 in mean change from baseline; −40%; *P* < 0.001). On measures of slope of progression, a statistically significant difference was observed on the ADCS‐ADL‐MCI between the aducanumab high‐dose group compared with placebo (annual decline of 2.1 points in the aducanumab high‐dose group versus 3.0 points in the placebo group, difference of 0.9; *P* < 0.05). There were no significant differences in the slope of decline for the aducanumab high‐dose group compared with placebo on the MMSE (difference of 0.5; *P* = 0.0503) or ADAS‐Cog13 (difference of −0.47; *P* = 0.34).

**FIGURE 5:**
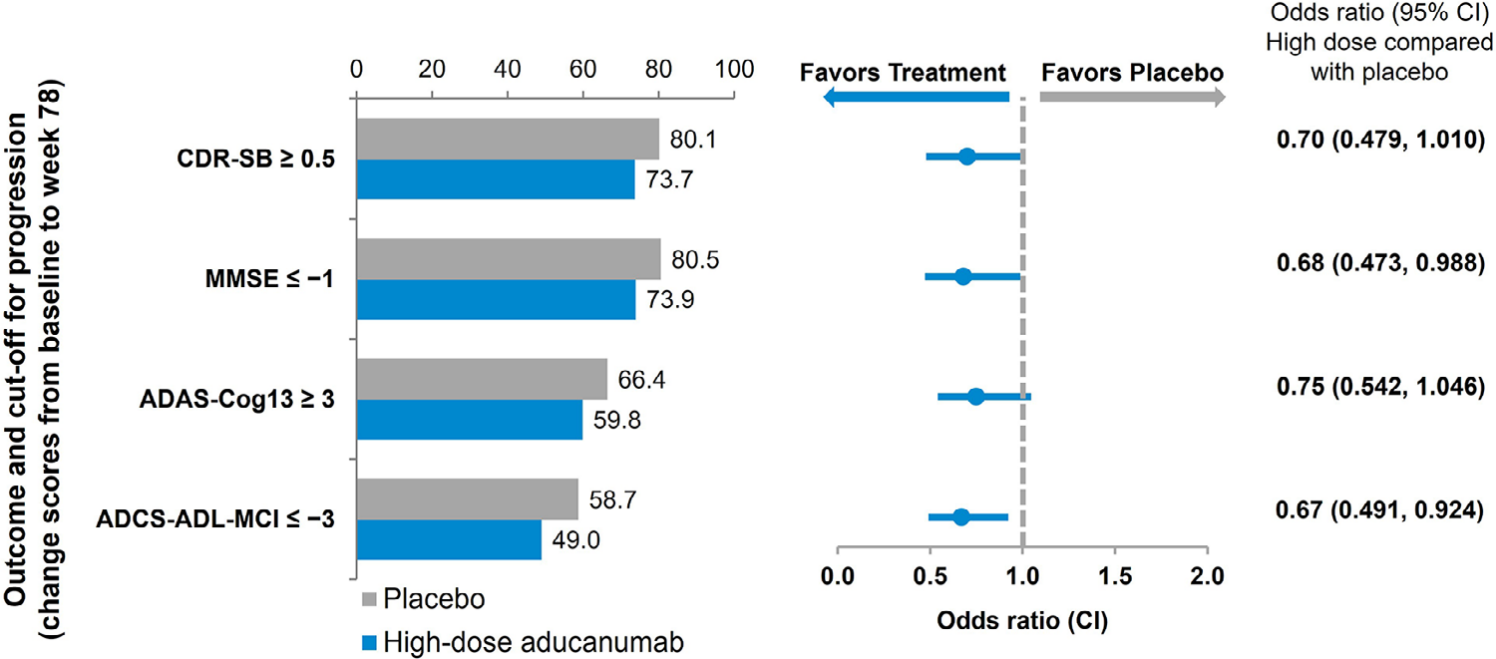
Odds ratio for progression in clinical endpoints at week 78: high‐dose versus placebo. The estimated percentage of progressors in the placebo group and high‐dose aducanumab group, as well as the odds ratio (95% confidence interval [CI]) of treatment with high‐dose compared with placebo in the opportunity to complete population for high‐dose aducanumab versus placebo are presented for Alzheimer's Disease Assessment Scale–Cognitive Subscale (13‐item) (ADAS‐Cog13); Alzheimer's Disease Cooperative Study–Activities of Daily Living Inventory (Mild Cognitive Impairment version) (ADCS‐ADL‐MCI); Clinical Dementia Rating Scale Sum of Boxes (CDR‐SB); and Mini‐Mental State Examination (MMSE).

A reduction in clinical decline at the level of individual participants was examined using a progression analysis and varying thresholds of decline from baseline (e.g., responder analysis). Clinical progression was defined as approximately 0.5 of the pooled standard deviation (SD) at baseline across all three treatment groups for each measure,^20^ corresponding to an increase of ≥0.5 points on the CDR‐SB, a decrease of ≥1 points on the MMSE, an increase of ≥3 points on the ADAS‐Cog13, and a decrease of ≥3 points on the ADCS‐ADL‐MCI. Based on these thresholds, 58% to 81% of participants in the placebo group had progressed at week 78. On each measure, participants treated with aducanumab were nearly 30% less likely to progress clinically, with odds ratios of 0.70 on the CDR‐SB (95% confidence interval [CI]: 0.479, 1.010), 0.68 on the MMSE (95% CI: 0.473, 0.988), 0.75 on the ADAS‐Cog13 (95% CI: 0.542, 1.046), and 0.67 on the ADCS‐ADL‐MCI (95% CI: 0.491, 0.924), each favoring aducanumab (Figure 5). A sensitivity analysis was conducted using a range of thresholds for each outcome, where clinical “progression” was defined as a worsening of the score more than the minimal score increment for each measure, and a similar trend was observed (Table S2 in supporting information).

The sustainability of the treatment effect was investigated using MMRM analysis by comparing the LTE early‐start group to the late‐start group. The differences in change from baseline on CDR‐SB score between the high‐dose aducanumab early‐ versus late‐start groups were −0.44 at week 78 (−25% difference; *P* < 0.05), −0.59 at week 106 (−23% difference; *P* < 0.05), and −0.51 at week 134 (−16% difference; *P* = 0.20) (Figure S2A in supporting information). Similar results were observed on the MMSE, ADAS‐Cog13, and ADCS‐ADL‐MCI (Figure S2B‐D in supporting information).

## 4 DISCUSSION

Aducanumab is an antibody targeting oligomeric and aggregated Aβ. Significant evidence of a dose‐dependent effect on the relevant amyloid (amyloid PET and cerebrospinal fluid [CSF] Aβ) and tau biomarkers (CSF p‐tau, CSF t‐tau, and plasma p‐tau) reported elsewhere support the conclusion that aducanumab modifies the underlying pathophysiology of AD.^14^ These results were consistent or directionally aligned with those observed in the aducanumab PRIME phase 1b study (NCT01677572).^21^, ^22^ Furthermore, group‐level correlation analyses based on data from EMERGE, ENGAGE, and PRIME demonstrated a correlation between treatment effects on Aβ PET and CDR‐SB, suggesting that a greater treatment effect on brain Aβ plaque levels was associated with a greater clinical benefit.^14^ In the EMERGE study, high‐dose aducanumab showed a statistically significant effect versus placebo on the change from baseline in CDR‐SB score at week 78 (primary endpoint) and on all secondary endpoints (MMSE, ADAS‐Cog13, ADCS‐ADL‐MCI). The difference between aducanumab and placebo reached nominal significance on the NPI‐10, a tertiary endpoint. The efficacy results were internally consistent, and it was previously reported that 79 of 80 subgroup comparisons across the five clinical endpoints showed treatment effects that favored aducanumab over placebo.^14^ The current study reports prespecified and post hoc analyses to further explore the clinical meaningfulness of the results.

The five clinical scales in EMERGE comprise a set of cognitive, functional, and neuropsychiatric instruments that, in combination, provide a comprehensive and validated evaluation of the AD clinical state and reflect the independent assessments of two skilled clinicians (a psychometrician and an independent CDR‐SB rater) along with participant and care partner input. These five clinical scales assess different aspects of cognition, daily function, and behavior with little overlap among measures (Figure S1 in supporting information). While the CDR‐SB, ADAS‐Cog13, and MMSE all measure aspects of cognition, they each capture distinct features of cognitive decline in AD and have unique psychometric properties, contributing to their lack of measurement overlap. The ADAS‐Cog13 measures aspects of memory, orientation, language, praxis, and visuospatial skills known to be compromised in AD.^23^ The CDR‐SB captures the global, clinical opinion of a rater after interviews with the care partner and participant and includes assessments missing in the ADAS‐Cog13 and MMSE: attention, working memory, and executive function. The MMSE, a brief cognitive screening tool validated in moderate‐ to late‐stage disease,^8^, ^19^ offers a sensitive measure that captures fluctuations in orientation and attention. These three unique measures of cognition provide, in aggregate, a comprehensive assessment of AD, when taken together with the ADCS‐ADL‐MCI and the NPI‐10, which capture the care partner's report of participant independence of daily activities and psychiatric symptoms, respectively. Notably, ADCS‐ADL‐MCI (14 concepts) and CDR‐SB (6 concepts) are among the clinical outcome assessments that have the most overlap with WMM concepts. Even though individual measures have limited overlap with the 42 concepts that matter most to people affected by AD, the combination of all five scales expands the coverage to relevance for 30 out of 42 concepts that are most important to people affected by AD.^13^

Overall, these results demonstrate that each scale contributes important information about the clinical state of the participant that is largely different from what is learned from the other scales. Consequently, when a treatment such as aducanumab demonstrates reduction of decline over 78 weeks on multiple clinical scales that represent a wide range of manifestations of disease progression, this suggests a broad treatment benefit. The mechanism of meaningful treatment benefit is supported by the earlier timing of the separation between high‐dose aducanumab versus placebo in two of the three cognitive domains (as early as week 50 for measures of Memory and Orientation) compared to the later finding for the functional domains (separation at week 78), consistent with the view that cognition drives function.

Odds ratios of progression were 0.75 or lower with aducanumab treatment. On CDR‐SB, more participants in the placebo (80.1%) than high‐dose (73.7%) groups progressed over 78 weeks, indicating that more participants in the high‐dose group had no decline (26.3% versus 19.9%). This is important for patients in the early symptomatic phase of the disease, when quality of life (QOL) remains good and early intervention allows more time to maintain the daily activities valued by the individual. These data are consistent with modeled projections from the EMERGE dataset that aducanumab treatment translated to a lower lifetime probability of transitioning to AD dementia, a lower lifetime probability of transitioning to institutionalization, delays in the median time to transition to AD dementia, and an incremental median time in the community of 1.32 years compared with standard of care.^24^

Slope analyses showed that the high‐dose aducanumab group declined on the CDR‐SB by 0.79 annually compared with a 1.09 annual decline in the placebo group. This implies that the magnitude of treatment effect would be greater than −0.39 for a treatment duration longer than 18 months. This is consistent with the expectations of a disease‐modifying treatment.^22^, ^25^ A sustained clinical response to longer‐term treatment suggests disease course–modifying properties. While the EMERGE LTE results must be interpreted with caution due to limited participant numbers and the exploratory nature of the analyses, the observed persistence in treatment effect over time with aducanumab therapy is consistent with a therapy that alters the course of the disease.^25^, ^26^, ^27^

From the real‐world perspective of what matters the most to people living with AD and their care partners,^28^ item‐level analyses of the CDR‐SB results demonstrated a consistent treatment effect across cognitive and functional domains that are relevant to changes in early AD. What is most meaningful to people living with AD and care partners at the early stages of disease is to maintain stable cognition and function and preserve independence for as long as possible.^10^ The decline observed for the five items within the ADAS‐Cog13 that are most sensitive to change (Orientation, Word Recall [both immediate and delayed], Word Recognition, and Number Cancellation tests) was 26% to 51% less in the high‐dose aducanumab group than in the placebo group. Across items of the ADCS‐ADL‐MCI, aducanumab treatment slowed the loss of functional independence. Consistent with previous reports, participants receiving placebo exhibited the greatest decline as assessed by the ADCS‐ADL‐MCI in higher‐order functional abilities that are most sensitive to change early in disease progression, such as making a meal, reading for more than 5 minutes, and using household appliances.^17^, ^29^ Aducanumab treatment preserved functional independence on these individual items by 65% to 91% over 18 months.

Neuropsychiatric symptoms are prevalent in early AD and strongly impact the QOL of patients and consequently of care partners.^30^, ^31^ With aducanumab treatment, the attenuation of psychiatric symptoms (87% reduction on the NPI‐10 scale) is accompanied by a decrease in care partner distress (84% reduction versus placebo on the overall NPI‐10 caregiver distress score). Reduction in care partner distress may translate into improved QOL for both the individual with AD and care partner and may delay nursing home placement.

Demonstration of a meaningful drug‐placebo difference depends in part on the decline observed in the placebo group. In EMERGE, the CDR‐SB worsened in the placebo group by only 1.74 points over 78 weeks, and approximately 20% of participants in the placebo group did not have any detectable worsening of CDR‐SB score (i.e., CDR‐SB change ≤0 over 78 weeks). The slowing observed on the CDR‐SB in the aducanumab high‐dose treatment group compared to placebo in EMERGE represents a first step toward establishing the threshold for clinically relevant effect sizes in an early AD population.

There is increasing agreement among regulatory agencies that demonstrating effects across multiple individual assessments would increase the persuasiveness of a clinically meaningful benefit in early‐stage disease.^5^, ^12^ Sponsors of trials in early AD use measurements from multiple domains or composite measures to directly assess clinically meaningful treatment effects.^3^, ^32^, ^33^, ^34^ For example, in CLARITY AD, data from cognitive, functional, QOL, and biomarker assessments offer converging evidence of a clinically meaningful effect from lecanemab treatment.^32^, ^35^ The integrated AD Rating Scale (iADRS), used as the primary outcomes in the donanemab trials, is a summary score of cognitive and functional items.^36^, ^37^

Clinically meaningful analysis approaches such as these using aducanumab data demonstrate how consistent clinical effects can be demonstrated across a variety of clinical outcomes, which span cognitive, functional, and neurobehavioral domains and differing analytical approaches. The findings are favorable toward aducanumab treatment across a variety of domains; they also align with content known to be noticeable and meaningful to people living with AD, care partners, and clinicians. Moreover, in this class of disease‐modifying treatments known to have substantial and durable effects on some biomarkers of AD,^14^, ^22^ it is expected that the clinical effects of slowing disease progression will persist or increase until delay in progression is attenuated and disease progression reasserts itself. Together the data and analyses presented here support the clinical meaningfulness of the effects observed with aducanumab treatment.

*Biogen licensed aducanumab from Neurimmune and has terminated that license. The rights to aducanumab have reverted to Neurimmune*

## CONFLICT OF INTEREST STATEMENT

J.M., H.M.B., M.N., J.O., and F.F. are employees and shareholders of Biogen. P.H., M.P., C.M., Y.T., L.Y., and S.B.H. were employees of Biogen at the time of this study and may own stock in Biogen. J.C. has provided consultation to Acadia, Acumen, ALZpath, Aprinoia, Artery, Biogen, Biohaven, BioXcel, Bristol Myers Squib, Eisai, Fosun, GAP Foundation, Janssen, Karuna, Kinoxis, Lighthouse, Lilly, Lundbeck, LSP/EQT, Merck, MoCA Cognition, New Amsterdam, Novo Nordisk, Optoceutics, Otsuka, Oxford Brain Diagnostics, Praxis, Prothena, ReMYND, Roche, Scottish Brain Sciences, Signant Health, Simcere, Sinaptica, TrueBinding, and Vaxxinity pharmaceutical, assessment, and investment companies. J.C. owns the copyright of the Neuropsychiatric Inventory. J.C. has stocks/options in Artery, Vaxxinity, Behrens, Alzheon, MedAvante‐Prophase, and Acumen. J.C. is supported by NIGMS grant P20GM109025; NIA grant R35AG71476; NIA R25 AG083721‐01; ADDF; Ted and Maria Quirk Endowment; and Joy Chambers‐Grundy Endowment. S.C. was an ENGAGE trial site investigator and an Aducanumab Steering Committee member. She is a consultant (no personal fees) to Alnylam, Biogen, Bristol Myers Squibb, Cassava Sciences, Cognivue, Cogstate, Eisai, Eli Lilly, INmune Bio, Lundbeck, Novartis, Novo Nordisk, Parexel, RetiSpec, Roche, and SciNeuro and receives research support (paid to institution) from AbbVie, Alnylam, AgeneBio, Alector, Alzheon, Anavex, Biogen, Cassava Sciences, Eisai, Eli Lilly, GSK, INmune Bio, Janssen, Novo Nordisk, RetiSpec, Roche, and UCB Biopharma. J.H. is an employee and shareholder in Scottish Brain Sciences. Additionally, he holds stock options in Neurotrack Inc. and is a joint holder of patents with MyCognition Ltd. J.J. is the owner and President of CognitionMetrics, L.L.C., which received fees from Biogen in consideration of scientific consulting services. C.J.M. was an ENGAGE trial site investigator and an Aducanumab Steering Committee member. She is supported by NIHR Biomedical Research Centre at UCLH and has acted as a consultant to Biogen, Eli Lilly, Roche, IONIS, Alector, Eisai, Alnylam, and Novartis. She has received research support from Biogen. A.P. reports personal fees from Acadia Pharmaceuticals, Avanir, Cadent Therapeutics, Functional Neuromodulation, Syneos, and BioXcel and grants to his institution from Avanir, Biogen, Biohaven, Eisai, Eli Lilly, Genentech/Roche, and Novartis. Author disclosures are available in the supporting information.

## CONSENT STATEMENT

The study was conducted in accordance with the Declaration of Helsinki and the International Conference on Harmonization and Good Clinical Practice guidelines and was approved by ethics committees or institutional review boards at each site. All patients provided written informed consent.

## Supporting information



Supporting Information

Supporting Information

Supporting Information

Supporting Information

Supporting Information
