# Supplementary material for: Evaluation of cognitive, functional, and behavioral effects observed in EMERGE, a phase 3 trial of aducanumab in people with early Alzheimer's disease
Source: Alzheimers Dement. 2025 Jun 22;21(6):e70224. doi: 10.1002/alz.70224 (PMC12183105; doi:10.1002/alz.70224)
Supplement: Supplementary file 1 — Supporting Information [file ALZ-21-e70224-s003.docx]

**Figure S1. Principal component analyses to determine the underlying constructs measured by the Clinical Dementia Rating – Sum of Boxes (CDR-SB), Mini-Mental State Examination (MMSE), Alzheimer’s Disease Assessment Scale–Cognitive Subscale (13-item) (ADAS-Cog 13), Alzheimer’s Disease Cooperative Study-Activities of Daily Living-Mild Cognitive Impairment (ADCS-ADL-MCI), and Neuropsychiatric Inventory Questionnaire (NPI-10).** Principal components (PCs) that explain 80% of the variance are reported for the **(A) placebo group baseline** data and **(B) change from baseline to week 78** data.


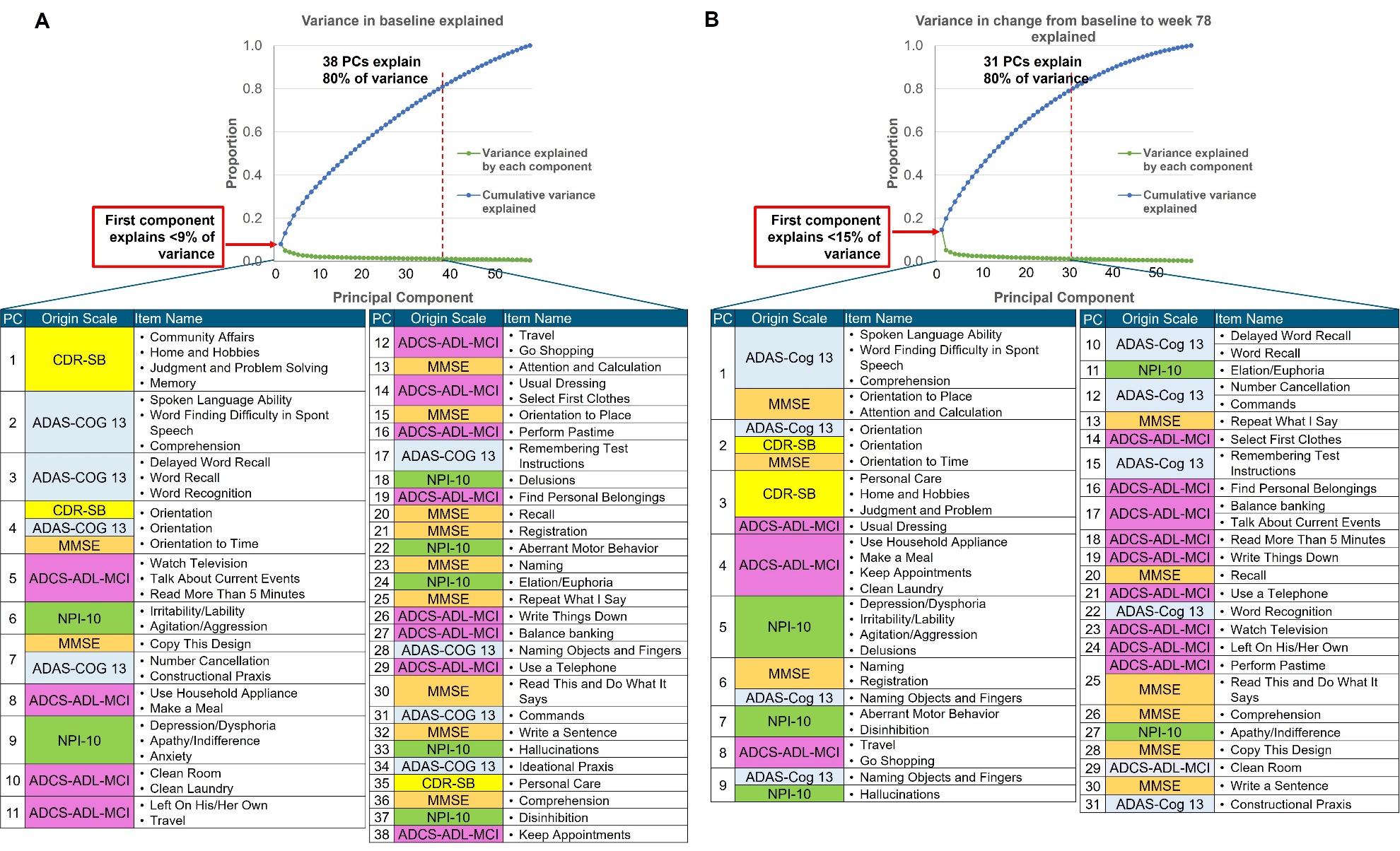


**Figure S2. Longitudinal change on the Clinical Dementia Rating – Sum of Boxes (CDR-SB), Mini-Mental State Examination (MMSE), Alzheimer’s Disease Assessment Scale–Cognitive Subscale (13-item) (ADAS-Cog 13), and Alzheimer’s Disease Cooperative Study-Activities of Daily Living-Mild Cognitive Impairment (ADCS-ADL-MCI).** Mean change from baseline **on the (A) CDR-SB, (B) MMSE, (C) ADAS-Cog 13, and (D) ADCS-ADL-MCI** at weeks 26, 50, 78, 106, and 134 are plotted with aducanumab high-dose treatment (solid line) and placebo (dotted). Dotted gray line indicates placebo group during the placebo-controlled period; solid blue line indicates high-dose aducanumab during the long-term extension. Higher scores on the CDR-SB and ADAS-Cog 13 indicate greater impairment. Lower scores on the MMSE and ADCS-ADL-MCI indicate greater impairment. SE, standard error.


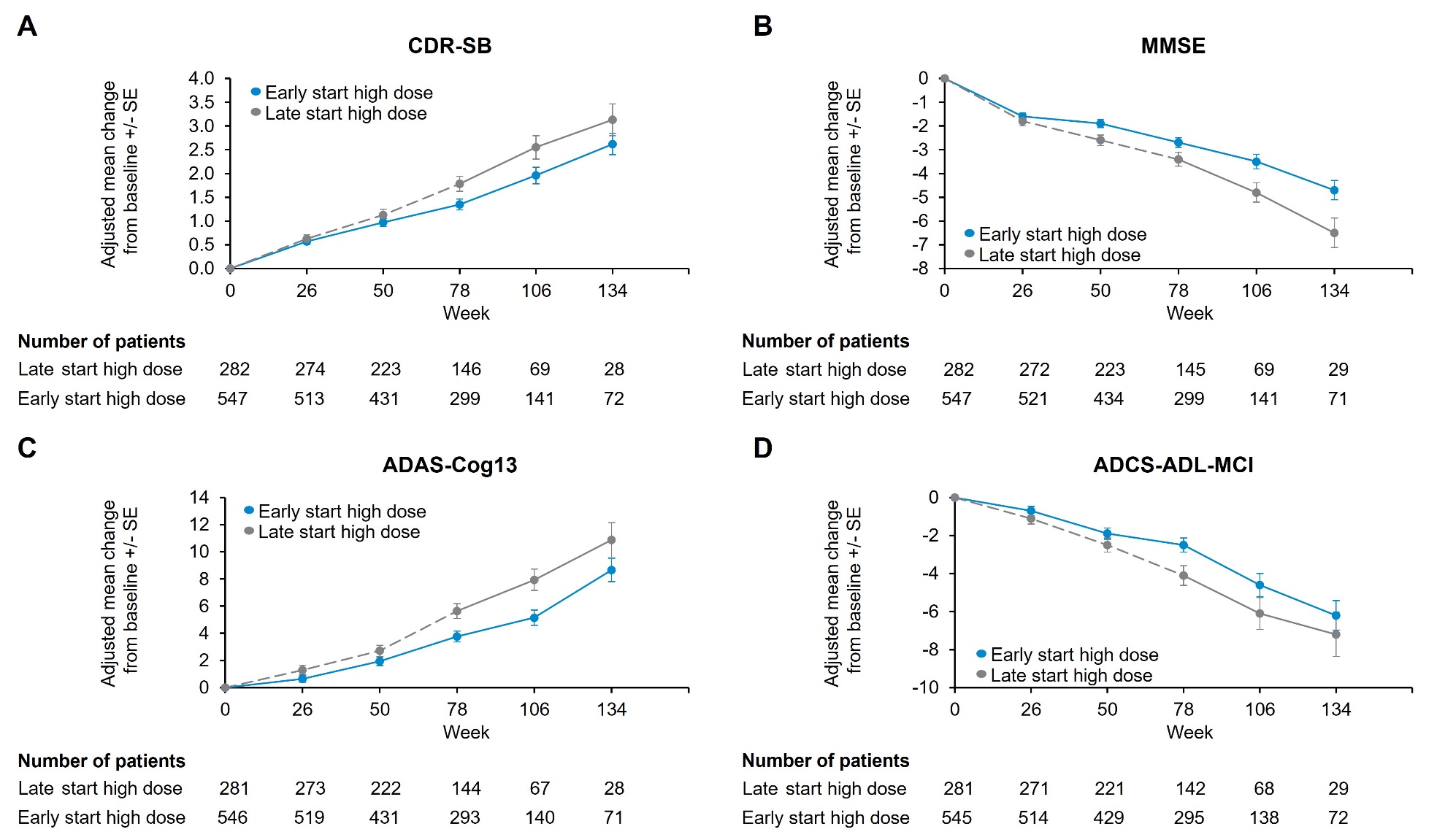


**Table S1. Baseline characteristics in the high-dose early-start and late-start groups in the long-term extension period**

|  | **Characteristics** | **Long-term extension period** | |  |
| --- | --- | --- | --- | --- |
|  |  | **High-dose late-start** | **High-dose early-start** |  |
|  |  | **(n=132)** | **(n=257)** |  |
|  | **Age in years, mean ± SD** | 70.2±7.0 | 70.6±7.6 |  |
|  | **Female, n (%)** | 73 (55) | 131 (51) |  |
|  | **Race, n (%)** |  |  |  |
|  | **Asian** | 8 (6) | 11 (4) |  |
|  | **White** | 111 (84) | 212 (83) |  |
|  | **Education years, mean ± SD** | 15.0±3.5 | 14.9±3.6 |  |
|  | **Alzheimer’s disease medications used, n (%)** | 71 (54) | 134 (52) |  |
|  | ***APOE* ε4, n (%)** |  |  |  |
|  | **Carriers** | 92 (70) | 164 (64) |  |
|  | **Noncarriers** | 40 (30) | 93 (36) |  |
|  | **Clinical stage, n (%)** |  |  |  |
|  | **MCI due to Alzheimer’s disease** | 116 (88) | 218 (85) |  |
|  | **Mild Alzheimer’s disease** | 16 (12) | 39 (15) |  |
|  | **RBANS delayed memory score, mean ± SD** | 58.0±13.8 | 61.5±14.0 |  |
|  | **CDR global score, n (%)** |  |  |  |
|  | **0.5** | 130 (99) | 257 (100) |  |
|  | **1** | 2 (2) | 0 (0) |  |
|  | **ITT** |  |  |  |
|  | **CDR-SB score, mean ± SD** | 2.5±0.9 | 2.4±1.0 |  |
|  | **MMSE score, mean ± SD** | 26.5±1.8 | 26.3±1.6 |  |
|  | **ADAS-Cog 13 score, mean ± SD** | 22.2±6.3 | 21.5±6.8 |  |
|  | **ADCS-ADL-MCI score, mean ± SD** | 42.8±4.7 | 42.9±5.5 |  |

NOTE: “High-dose late-start” refers to patients who received placebo during the placebo-controlled period and switched to aducanumab high-dose during the LTE period. “High-dose early-start” refers to patients who received aducanumab high-dose during both the placebo-controlled and LTE period.

Data are mean ± SD or n (%).

Abbreviations: ADAS-Cog 13, Alzheimer’s Disease Assessment Scale–Cognitive Subscale (13-item); ADCS-ADL-MCI, Alzheimer’s Disease Cooperative Study-Activities of Daily Living-Mild Cognitive Impairment; ApoE, apolipoprotein E; CDR-SB, Clinical Dementia Rating – Sum of Boxes; ITT, intention to treat; LTE, long-term extension; MCI, mild cognitive impairment; MMSE, Mini-Mental State Examination; RBANS, Repeatable Battery for Assessment of Neuropsychological Status; SD, standard deviation.

**Table S2.** **Progression analyses on the CDR-SB, MMSE, ADAS-Cog 13, and ADCS-ADL-MCI using a range of thresholds for each outcome**

|  | **Scale** | **Cut-off** | **Estimated percentage of progressors: placebo** | **Odds ratio (95% CI)**  **High dose compared with placebo** |  |
| --- | --- | --- | --- | --- | --- |
|  | **CDR-SB** | **≥ 0.5** | 80.1 | 0.70 (0.479, 1.010) |  |
|  |  | **≥ 1.0** | 66.1 | 0.71 (0.510, 0.983) |  |
|  |  | **≥ 1.5** | 52.1 | 0.76 (0.556, 1.043) |  |
|  | **MMSE** | **≤ −1** | 80.5 | 0.68 (0.473, 0.988) |  |
|  |  | **≤ −2** | 68.6 | 0.76 (0.544, 1.057) |  |
|  |  | **≤ −3** | 52.7 | 0.86 (0.631, 1.181) |  |
|  | **ADAS-Cog13** | **≥ 2** | 68.8 | 0.88 (0.624, 1.235) |  |
|  |  | **≥ 3** | 66.4 | 0.75 (0.542, 1.046) |  |
|  |  | **≥ 4** | 57.0 | 0.86 (0.630, 1.187) |  |
|  | **ADCS-ADL-MCI** | **≤ −1** | 68.4 | 0.68 (0.485, 0.948) |  |
|  |  | **≤ −2** | 63.8 | 0.74 (0.537, 1.022) |  |
|  |  | **≤ −3** | 58.7 | 0.67 (0.491, 0.924) |  |
|  |  | **≤ −4** | 52.8 | 0.63 (0.458, 0.858) |  |

Abbreviations: ADAS-Cog 13, Alzheimer’s Disease Assessment Scale–Cognitive Subscale (13-item); ADCS-ADL-MCI, Alzheimer’s Disease Cooperative Study-Activities of Daily Living-Mild Cognitive Impairment; CDR-SB, Clinical Dementia Rating – Sum of Boxes; CI, confidence interval; MMSE, Mini-Mental State Examination.
