# Supplementary material for: Evaluation of cognitive, functional, and behavioral effects observed in EMERGE, a phase 3 trial of aducanumab in people with early Alzheimer's disease
Source: Alzheimers Dement. 2025 Jun 22;21(6):e70224. doi: 10.1002/alz.70224 (PMC12183105; doi:10.1002/alz.70224)
Supplement: Supplementary file 3 — Supporting Information [file ALZ-21-e70224-s001.docx]

**Supporting Information**

Supplement to: Cummings J, et al. Evaluation of cognitive, functional and behavioral effects observed in EMERGE, a phase 3 clinical trial of aducanumab in participants with early Alzheimer’s disease

**File S1. Trial Design**

Aducanumab or placebo was administered via intravenous infusion every 4 weeks over 76 weeks (20 doses total). Participants were stratified by apolipoprotein E (*APOE*) *ε4* carrier status and were randomized (1:1:1) to receive low-dose aducanumab, high-dose aducanumab, or placebo. The dose in the low-dose group was titrated to a target dose of 3 mg/kg (*APOE ε4+*) or 6 mg/kg (*APOE ε4-*). The dose in the high-dose group was titrated to a target dose of 6 mg/kg (*APOE ε4+*) or 10 mg/kg (*APOE ε4-*) prior to protocol amendments. To maximize the dose-dependent effect of aducanumab [14], the target dose for ApoE ε4+ carriers in the high-dose regimen was increased from 6 to 10 mg/kg in protocol version 4 (PV4; approved on March 24, 2017, and implemented over approximately 18 months across sites).

The final analysis (intention to treat [ITT]) of all patient data from EMERGE excluding efficacy assessments performed after the futility announcement included 1638 patients (548 placebo, 543 low-dose aducanumab, 547 high-dose aducanumab) [13] (**Figure 1**). The opportunity to complete analysis (OTC; ITT population that had the opportunity to complete week 78 prior to futility announcement) included 982 patients (313 placebo, 329 low-dose aducanumab, 340 high-dose aducanumab). Following the 78-week placebo-controlled (PC) study period, an optional long-term extension (LTE) period on active treatment evaluated the longer-term safety and efficacy of aducanumab. During the LTE, participants and site personnel remained blinded to the dose assignment during the PC period of the study.

All patients provided written informed consent. The study was conducted in accordance with the Declaration of Helsinki and the International Conference on Harmonisation and Good Clinical Practice guidelines. Ethics committee and institutional review board approval for the study protocol and all amendments were obtained for each participating site.
