# Supplementary material for: Evaluation of cognitive, functional, and behavioral effects observed in EMERGE, a phase 3 trial of aducanumab in people with early Alzheimer's disease
Source: Alzheimers Dement. 2025 Jun 22;21(6):e70224. doi: 10.1002/alz.70224 (PMC12183105; doi:10.1002/alz.70224)
Supplement: Supplementary file 4 — Supporting Information [file ALZ-21-e70224-s002.docx]

**Supporting Information**

Supplement to: Cummings J, et al. Evaluation of cognitive, functional and behavioral effects observed in EMERGE, a phase 3 clinical trial of aducanumab in participants with early Alzheimer’s disease

**File S2. Clinical outcome measures**

These cognitive, functional and neuropsychiatric measures reflect disease manifestations and symptom severity, and assess the ability of a potential therapeutic intervention to slow progression of clinical signs and symptoms. The primary outcome measure, Clinical Dementia Rating – Sum of Boxes (CDR-SB), assesses both cognition and function in Alzheimer’s disease and is the “sum of boxes” score of the six CDR domains (Orientation, Memory, Judgment and Problem Solving, Community Affairs, Home and Hobbies, and Personal Care). The CDR is a composite instrument in that it includes both tests of cognitive ability that are administered to the patient and semistructured interviews of the patient and caregiver to determine the patient’s clinical and functional status. A clinical rater assigns a score based on his/her clinical judgment after a thorough semi-structured interview with the patient and a reliable informant (family member or care partner) [17]. The secondary (Mini-Mental State Examination [MMSE], Alzheimer’s Disease Assessment Scale–Cognitive Subscale [13-item] [ADAS-Cog 13], Alzheimer’s Disease Cooperative Study-Activities of Daily Living-Mild Cognitive Impairment [ADCS-ADL-MCI]) and tertiary (Neuropsychiatric Inventory Questionnaire [NPI-10]) efficacy outcome measures all complement the CDR-SB, and each provides important and largely independent clinical information about patients, with minimal overlap across measures. The MMSE assesses cognitive decline and is widely used by physicians in clinical practice due to its simplicity and speed of administration [18]. ADAS-Cog 13 comprises both cognitive tasks and clinical ratings of cognitive performance [19,20]. The scale captures items such as word recall, word recognition, orientation, with a measure for delayed word recall and concentration/distractibility, and is more complex and subjective than the MMSE [21]. ADCS-ADL-MCI is a measure of daily activities, which consists of 17 instrumental items (e.g. shopping, preparing meals, using household appliances) and one basic item (getting dressed). It reflects the informant’s observations of more nuanced changes in the patient’s functional ability, especial in early disease [19]. The NPI-10 is an informant-based assessment that systematically indexes the presence, frequency and severity of 10 neuropsychiatric symptoms such as agitation/aggression, apathy, anxiety and depression/dysphoria; associated caregiver distress with symptoms is also measured [22]. This wide array of cognitive, functional, and neuropsychiatric measures provides a comprehensive assessment of the Alzheimer’s disease state, with demonstrated sensitivity to change as the disease progresses, and collectively reflect three independent sources of information: the patient, caregiver, and independent clinical assessors.
