# Supplementary material for: Evaluation of cognitive, functional, and behavioral effects observed in EMERGE, a phase 3 trial of aducanumab in people with early Alzheimer's disease
Source: Alzheimers Dement. 2025 Jun 22;21(6):e70224. doi: 10.1002/alz.70224 (PMC12183105; doi:10.1002/alz.70224)
Supplement: Supplementary file 5 — Supporting Information [file ALZ-21-e70224-s005.docx]

**Supporting Information**

Supplement to: Cummings J, et al. Evaluation of cognitive, functional and behavioral effects observed in EMERGE, a phase 3 clinical trial of aducanumab in participants with early Alzheimer’s disease

**File S3. Responder analysis and post-hoc progression analyses**

A responder analysis was pre-specified in the statistical analysis plan based on two definitions: first, responders had CDR-SB change ≤0.5 from baseline at week 78; second, responders had CDR-SB change ≤1.5 from baseline at week 78. The dichotomized response, responder vs. nonresponder, was modeled using a logistic regression with the following covariates: treatment group, baseline CDR-SB, baseline MMSE, AD symptomatic medication use at baseline (yes/no), world region, and laboratory *APOE ε4* status (carrier/non-carrier). Because the amount of data missing at week 78 was high in the ITT population due to early administrative terminations, data from participants with the OTC the week 78 visit prior to the futility declaration were used for these analyses.

Post-hoc progression analyses in CDR-SB, MMSE, ADAS-Cog13 and ADCS-ADL-MCI assessed aducanumab’s effect in delaying clinical decline at the individual patient-level. For these analyses progression was defined operationally as approximately 0.5 of the pooled SD at EMERGE baseline for each measure [15], corresponding to an increase of ≥0.5 points on the CDR-SB, a decrease of ≥1 point on the MMSE, an increase of ≥3 points on the ADAS-Cog13, and a decrease of ≥3 points on the ADCS-ADL-MCI. The odds ratios for “progression” in the OTC population for high-dose vs placebo were calculated for these cut-offs. A CDR-SB progression analysis, in which a progressor is defined as someone with an increase of ≥1 point in CDR-SB at week 78, is equivalent to the pre-specified responder analysis where response is defined as an increase ≤0.5 points.

A progression analysis was used to examine the clinical meaningfulness of the benefit of aducanumab on the individual patient using the concept of minimal clinically important difference (MCID), which represents the smallest change in score in a patient- or clinician-reported instrument that is perceived by a patient, caregiver, or clinician as beneficial, and thereby clinically meaningful. MCID is derived using two main methodologies. In anchor-based methods, changes seen in an instrument score are compared (“anchored”) to other clinical changes deemed to represent a known “meaningful change.” Distribution-based methods use the statistical distribution properties of the population studied placebo group or natural history cohort to derive a threshold for interpretation. In the absence of an established clinically meaningful “deteriorator threshold” derived from anchor based analytic methods, examination of the population distribution statistics offers a robust starting point. (Andrews et al. 2019) This method allowed us to establish that each of the clinical endpoints measured a numerical advantage in favor of aducanumab.
